# Paracrine cross-talk between skeletal muscle and macrophages in exercise by PGC-1α-controlled BNP

**DOI:** 10.1038/srep40789

**Published:** 2017-01-16

**Authors:** Regula Furrer, Petra S. Eisele, Alexander Schmidt, Markus Beer, Christoph Handschin

**Affiliations:** 1 Biozentrum, Division of Pharmacology/Neurobiology, University of Basel, CH-4056 Basel, Switzerland; 2 Biozentrum, Proteomics Core Facility, University of Basel, CH-4056 Basel, Switzerland

## Abstract

Activation of resident and infiltrating immune cells is a central event in training adaptation and other contexts of skeletal muscle repair and regeneration. A precise orchestration of inflammatory events in muscle fibers and immune cells is required after recurrent contraction-relaxation cycles. However, the mechanistic aspects of this important regulation remain largely unknown. We now demonstrate that besides a dominant role in controlling cellular metabolism, the peroxisome proliferator-activated receptor γ co-activator 1α (PGC-1α) also has a profound effect on cytokine expression in muscle tissue. Muscle PGC-1α expression results in activation of tissue-resident macrophages, at least in part mediated by PGC-1α-dependent B-type natriuretic peptide (BNP) production and secretion. Positive effects of exercise in metabolic diseases and other pathologies associated with chronic inflammation could accordingly involve the PGC-1α-BNP axis and thereby provide novel targets for therapeutic approaches.

Skeletal muscle plasticity in response to acute bouts of exercise involves sterile tissue inflammation. A tightly coordinated activation of tissue-resident and infiltrating immune cells is instrumental for proper muscle repair and regeneration after these recurring contraction-relaxation cycles. During this process, macrophages polarize into the M1-type inducing a pro-inflammatory cytokine profile to stimulate tissue clean-up^1^. This is followed by a second wave of M2 macrophage polarization that is linked to a predominantly anti-inflammatory milieu to boost tissue repair and regeneration^1^. In contrast to the transient inflammation after exercise, a persistent, low-grade elevation of pro-inflammatory factors, both locally in tissues as well as systemically, is closely associated with a number of chronic diseases, including the metabolic syndrome or cardiovascular pathologies^1^. Exercise combined with other life-style interventions efficiently prevents or ameliorates many of these diseases^2^,^3^,^4^. In response to exercise, skeletal muscle cells secrete various factors, so-called myokines, which elicit responses in an auto-, para- or endocrine manner^5^,^6^,^7^. Although several factors are known to be secreted by exercised muscles, our understanding of the cross-talk between muscle fibers and other cell types in exercise or disease remains rudimentary^7^. In particular, it is unclear whether immune cell activation in muscle tissue is a secondary event triggered by fiber damage or if muscle fibers exert direct control on tissue-resident macrophages.

The peroxisome proliferator-activated receptor γ (PPARγ) co-activator 1α (PGC-1α) is a central regulator of endurance training adaptation in skeletal muscle. PGC-1α induces genes involved in mitochondrial biogenesis, oxidative phosphorylation, vascularization and the contractile apparatus to enable a higher endurance capacity by co-activating an array of nuclear receptors and other transcription factors in a poorly understood complex transcriptional network^8^,^9^. In addition to the effects on energy metabolism in response to exercise, PGC-1α also has an immunomodulatory role^1^. In cultured muscle cells, overexpression of PGC-1α exerts a dampening of nuclear factor κB activity and consequently a suppression of pro-inflammatory gene expression upon treatment with tumor necrosis factor α (TNFα), saturated fatty acids or specific activators of the toll-like receptors 1/2, 4 and 6/2^10^. In contrast, specific PGC-1α overexpression in muscle fibers *in vivo* was not able to reduce the strong, acute inflammation induced by TNFα or bacterial lipopolysaccharide (LPS), indicating a cross-talk of different cell types to overcome the cell autonomous anti-inflammatory effect of PGC-1α in muscle tissue^11^. These findings imply a multifaceted role for muscle PGC-1α to modulate local inflammation that extends beyond cell autonomous effects with important implications for our understanding of muscle cell plasticity in exercise and disease.

In the present study, we therefore explored the effects of exercise and PGC-1α in skeletal muscle *in vivo* on inflammation and in particular the possible cross-talk between muscle cells and macrophages. By combining *in vivo* experiments with *in vitro* sectretome investigations, we identified B-type natriuretic peptide (BNP) as a novel PGC-1α-dependent myokine that mediates cross-talk with tissue macrophages in skeletal muscle.

## Results

### Exercise and PGC-1α overexpression increase pro- and anti-inflammatory cytokines in skeletal muscle

**Figure 1:**
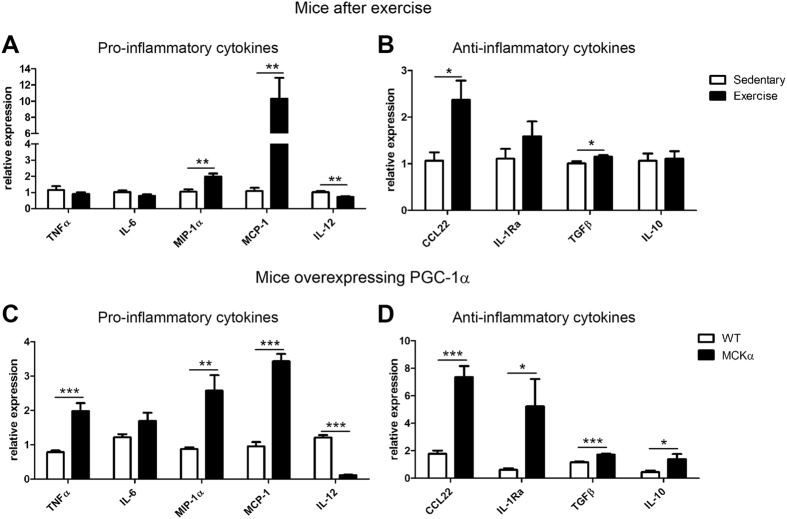
Exercise and PGC-1α overexpression increase pro- and anti-inflammatory cytokines in skeletal muscle. (**A–D**) Relative expression levels of pro- and anti-inflammatory cytokines were analyzed by RT-PCR in quadriceps muscle after exercise (panel A and B) and in MCKα mice (panel C and D). Values represent the mean of at least 6 animals +SEM. *P < 0.05; **P < 0.01; ***P < 0.001; exercise versus sedentary, MCKα versus WT.

As a first step to investigate muscle-immune cell cross-talk, expression levels of pro-inflammatory cytokines associated with M1 macrophage activation (i.e. TNFα, interleukin 6 (IL-6), macrophage inflammatory protein 1α (MIP-1α), monocyte chemoattractant protein 1 (MCP-1) and IL-12) and anti-inflammatory cytokines associated with M2 activation (i.e. C-C motif chemokine 22 (CCL22), IL-1Ra, transforming growth factor β (TGFβ) and IL-10) were determined in quadriceps muscle of mice after a bout of endurance exercise to exhaustion. A change in the expression levels of some of these pro- and anti-inflammatory genes was indeed observed in response to exercise (Fig. 1A and B).

We then mapped the cytokine environment in the quadriceps muscle of sedentary PGC-1α muscle-specific transgenic mice (MCKα) and their wild-type littermates (WT) to study whether muscle-specific overexpression of PGC-1α induces a similar cytokine profile as exercise. In MCKα mice, expression of pro-inflammatory cytokines TNFα, MIP-1α and MCP-1 was elevated whereas IL-6 mRNA expression was similar to WT mice (Fig. 1C). As reported previously^11^, IL-12 levels were strongly suppressed by PGC-1α overexpression. In contrast to the mixed M1 cytokine expression pattern, all of the tested M2 cytokines were significantly augmented by PGC-1α overexpression (Fig. 1D). Our data demonstrate that exercise and PGC-1α alone induce the expression of both pro- and anti-inflammatory cytokines in skeletal muscle. Importantly, even though a strong anti-inflammatory pattern of cytokine expression correlating to an M2-type polarization of macrophages is observed, the increase in some pro-inflammatory cytokines suggests that muscle PGC-1α could generally prime macrophages for rapid action after an exercise bout, in which an M1 to M2 transition has to be tightly orchestrated to ensure proper repair and regeneration.

### Cell autonomous and non-autonomous effects of muscle PGC-1α *in vitro*

**Figure 2:**
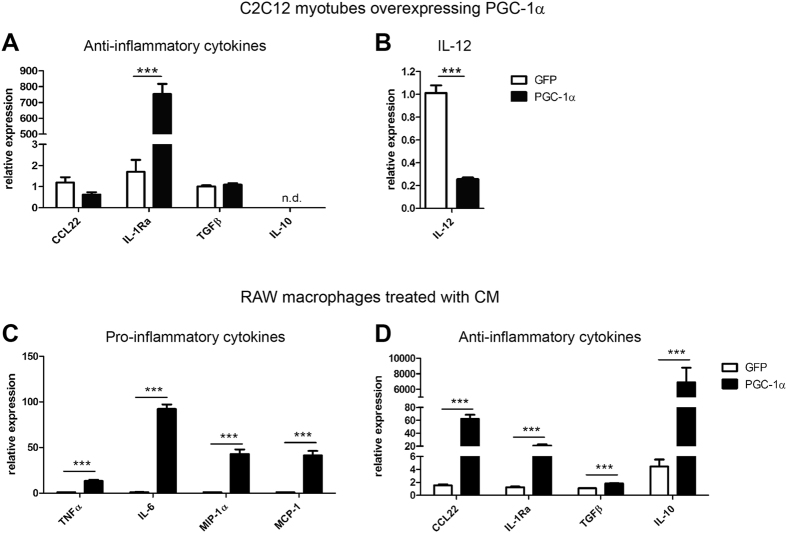
Cell autonomous and non-autonomous effects of muscle PGC-1α *in vitro.* (**A,B**) mRNA expression of anti-inflammatory cytokines (**A**) and IL-12 (**B**) in C2C12 myotubes adenovirally overexpressing PGC-1α or GFP. (**C,D**) RAW macrophages were treated with conditioned medium of C2C12 myotubes overexpressing PGC-1α or GFP and expression levels of pro- (**C**) and anti-inflammatory (**D**) cytokines were determined. Values represent the mean of at least 3 independent experiments +SEM. ***P < 0.001; PGC-1α versus GFP. See also Figure S1.

Due to the high complexity of skeletal muscle tissue *in vivo*, it was not possible to distinguish muscle-derived cues from those produced and secreted by cells of the immune system in our experimental settings. We therefore analyzed cytokine mRNA levels in cultured C2C12 myotubes overexpressing either PGC-1α or GFP. Transcript levels of IL-10 were below the detection limit of the qPCR assay and thus most likely derived from immune cells *in vivo*. CCL22 and TGFβ were detectable, albeit at very low amounts. These genes were however not regulated by PGC-1α in cultured muscle cells as observed in muscle tissue *in vivo* (Fig. 2A). These results imply that *in vivo,* immune rather than muscle cells are the main source of these cytokines. In contrast, the induction of IL-1Ra and the strong suppression of IL-12 observed in MCKα mice *in vivo* were recapitulated in the cellular system upon PGC-1α overexpression (Fig. 2B). Thus, the very low IL-12 levels detected in muscle of transgenic mice are likely due to very low expression of this cytokine in muscle cells themselves.

To dissect the role of muscle PGC-1α on pro- and anti-inflammatory cytokine expression of macrophages, RAW macrophages were treated with conditioned medium (CM) of C2C12 myotubes overexpressing PGC-1α (or GFP as control). After 4 hours, both M1 and M2 cytokine expression was augmented in RAW macrophages (Fig. 2C and D). These results show that *in vitro*, muscle PGC-1α increases the production and secretion of soluble factors that subsequently activate a pro- and an anti-inflammatory response in macrophages.

### PGC-1α induces the secretion of proteins from muscle cells to activate macrophages

PGC-1α regulates a number of myokines in skeletal muscle^7^. The effects of such known myokines on M1 and M2 cytokine expression were tested by treating RAW macrophages with irisin, IL-15, brain-derived neurotrophic factor (BDNF), the chemokine C-X-C motif ligand 1 (CXCL1), CXCL2 or CXCL5 (the latter three are mouse IL-8 homologues). These different myokines only induced mild changes in M1 and M2 expression levels and were thereby not likely to account for the strong effects observed after CM treatment (Fig. S1A).

**Figure 3:**
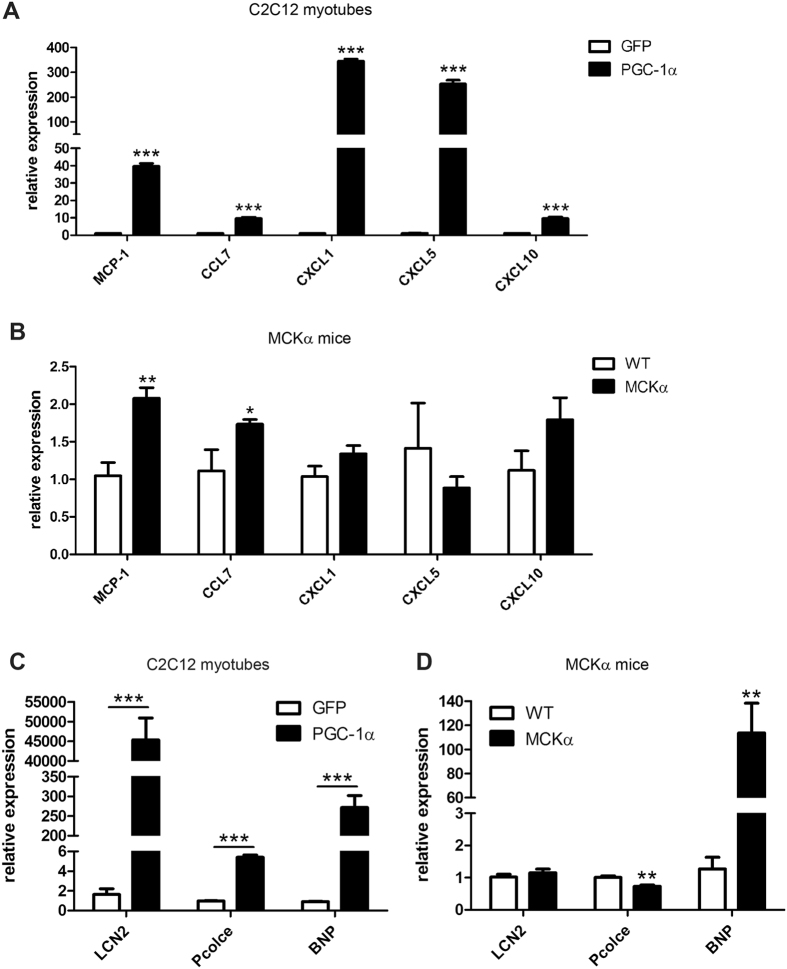
PGC-1α induces the secretion of proteins from muscle cells to activate macrophages. (**A,B**) mRNA expression levels of chemokines identified in the CM of C2C12 myotubes overexpressing PGC-1α by mass spectrometry were validated in C2C12 myotubes overexpressing PGC-1α (**A**) and MCKα mice (**B**). (**C,D**) Other identified factors from the CM potentially involved in the muscle-macrophage cross-talk were validated by RT-PCR. mRNA expression levels of LCN2, Pcolce and BNP were determined in C2C12 myotubes adenovirally overexpressing PGC-1α or GFP (**C**) and in WT and MCKα mice (**D**). Values represent the mean of at least 3 independent experiments or at least 6 animals +SEM. *P < 0.05; **P < 0.01; ***P < 0.001; PGC-1α versus GFP, MCKα versus WT.

To identify other factors secreted upon PGC-1α expression, mass spectrometry of CM was performed to screen the muscle cell secretome in an unbiased manner. This proteomic approach identified several proteins that potentially contribute to the cross-talk between muscle cells and macrophages (Table S2). Several chemokines (CCL2/MCP-1, CCL7, CXCL1, CXCL5 and CXCL10), of which an immunomodulatory role is already known, were among the candidate proteins (Table 1). Their relative gene expression was verified in C2C12 myotubes and as expected, the transcript levels of these chemokines were strongly induced (Fig. 3A). *In vivo* however, only MCP-1 and CCL7 expression was higher in MCKα mice compared to WT animals (Fig. 3B).

**Figure 4:**
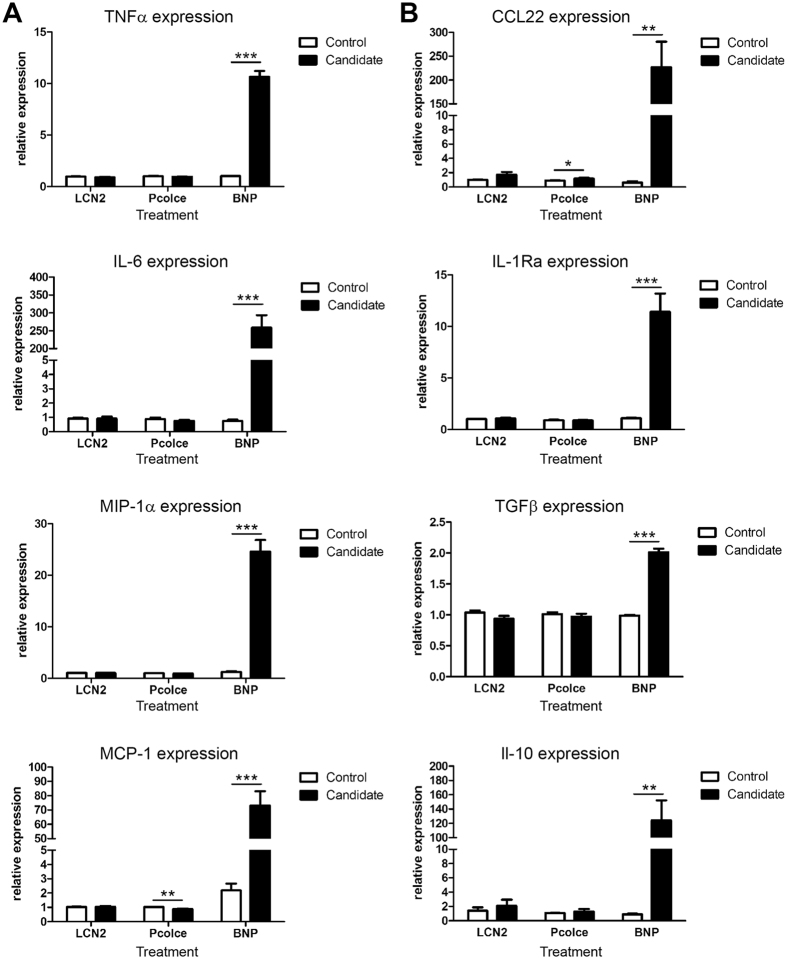
BNP increased the expression of pro- and anti-inflammatory cytokines in RAW macrophages. (**A,B**) RAW macrophages were treated for 4 h with LCN2, Pcolce or BNP. Relative expression of pro- (**A**) and anti-inflammatory (**B**) cytokines was determined by RT-PCR. Values represent the mean of at least 3 independent experiments +SEM. **P < 0.01; ***P < 0.001; candidate versus control. See also Figure S2.

In addition to these chemokines, our analysis of CM from PGC-1α-overexpressing myotubes revealed other, non-chemokine proteins (Table 1). Among these, the 3 most interesting candidate proteins within the secretome were lipocalin 2 (LCN2), procollagen C-endopeptidase enhancer 1 (Pcolce) and B-type (or brain) natriuretic peptide (BNP, gene name *Nppb*). The induction of these candidate genes by PGC-1α was validated in cultured myotubes and mice (Fig. 3C and D). In contrast to the elevation of all three genes in C2C12 myotubes overexpression PGC-1α, only BNP expression was enhanced in muscle-specific PGC-1α transgenic mice *in vivo*. To compare whether these candidates would elicit a similar response as CM from PGC-1α-overexpressing myotubes, RAW macrophages where directly treated with recombinant LCN2, Pcolce or BNP. Of these three candidates, BNP pronouncedly increased the expression of M1 and M2 cytokines, whereas LCN2 and Pcolce had no effect (Fig. 4A and B). Importantly, BNP-induced cytokine expression was dose-dependent and both qualitatively and quantitatively closely resembled CM treatment (Fig. S2). These results indicate that BNP plays an important role in stimulating the expression of M1 and M2 cytokines in macrophages.

### BNP is a PGC-1α-dependent myokine

Our results show that BNP may be responsible for para-crine effects of PGC-1α on macrophages. The high transcript levels of BNP in C2C12 myotubes overexpressing PGC-1α strongly suggest that BNP is indeed regulated by PGC-1α. To further elucidate the epistasis between these two genes, the BNP promoter region was scanned for potential PGC-1α response elements based on previous work on the PGC-1α-controlled transcriptional network in muscle cells^12^. The activator protein-1 (AP-1) transcription factor protein complex, which binds to Fos-Jun-like motifs, has two predicted binding sites in the genomic region flanking the BNP transcriptional start site (TSS), and is co-activated by PGC-1α^12^. Accordingly, siRNA-based silencing of Fos and Jun strongly reduces the ability of PGC-1α to induce BNP expression^12^.

**Figure 5:**
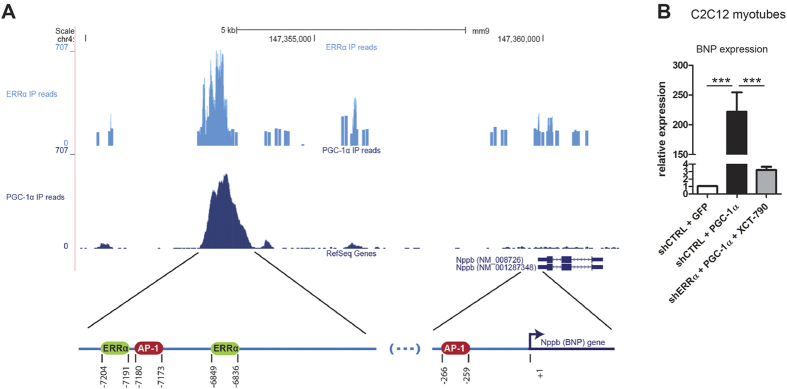
BNP is a PGC-1α-dependent myokine. (**A**) Overlapping ChIP-Seq peaks of ERRα (light blue) and PGC-1α (dark blue) are located ~7 kb upstream of the *Nppb* (BNP) RefSeq transcripts and depicted as read densities. Predicted binding sites for AP-1 and ERRα are represented by red and light green circles, respectively, and their genomic positions are referred to the BNP transcription start site (TSS) (**B**) Relative expression levels of BNP in response to PGC-1α overexpression and shERRα knock-down, in addition to the inverse agonist XCT-790 were determined by RT-PCR. Values represent the mean of at least 3 independent experiments +SEM. ***P < 0.001.

Several PGC-1α peaks, located upstream of the BNP TSS were identified using ChIPseq data of global PGC-1α DNA recruitment to the mouse genome in muscle cells^12^ (Fig. 5A). Intriguingly, the absence of a PGC-1α peak in the proximal promoter region of BNP suggests that the AP-1 binding site in this region is indirectly activated by PGC-1α. Within the most prominent peak that is located ~7 kilo base (kb) upstream of the BNP TSS, two binding motifs for estrogen-related receptor α (ERRα, ESRRA motif) and one binding site for AP-1 were predicted. Analyses of ERRα ChIP-Seq data^13^ revealed an overlap of a PGC-1α and an ERRα peak at this genomic location, strongly suggesting co-recruitment of these two proteins. Potent inhibition of ERRα with shRNA and the inverse agonist XCT790^14^ resulted in a marked reduction of PGC-1α-mediated BNP expression establishing a functional relationship between PGC-1α and ERRα in the control of BNP transcription (Fig. 5B). Collectively, these results suggest that PGC-1α regulates BNP gene expression in conjunction with AP-1 and ERRα, similarly to what has been described for vascular endothelial growth factor and other PGC-1α target genes^8^.

### BNP is regulated by PGC-1α and exercise in muscle *in vivo*

**Figure 6:**
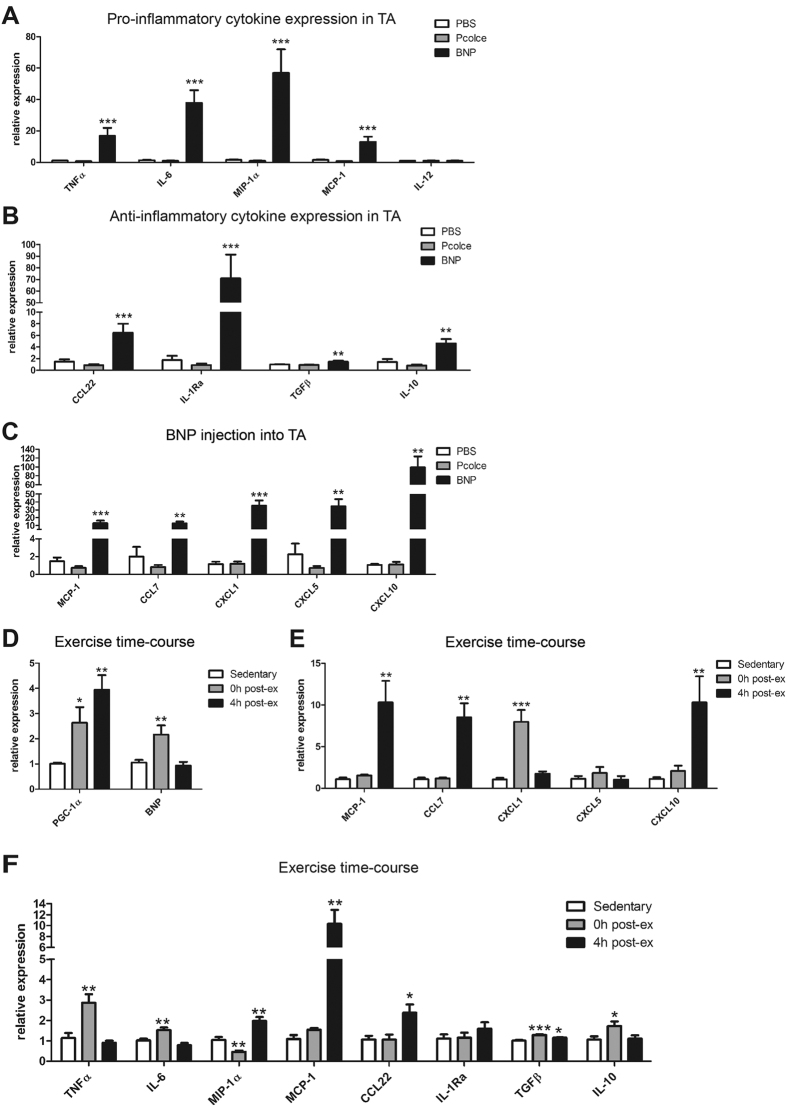
BNP is regulated by PGC-1α and exercise in muscle *in vivo.* (**A,B**) Four hours after *i.m.* injection of BNP, Pcolce or PBS into TA muscle, mRNA expression of pro- (**A**) and anti-inflammatory (**B**) cytokines was determined. (**C**) Chemokine expression levels were also measured after *i.m*. injections and were largely increased. (**D–F**) An exercise test to exhaustion was performed and quadriceps muscle was analyzed immediately (0 h post-exercise) and 4 h post-exercise. Time courses of PGC-1α and BNP (**D**), chemokines and pro- and anti-inflammatory cytokines (**F**) expression were determined. Values represent the mean of at least (**E**) 6 animals +SEM. *P < 0.05; **P < 0.01; ***P < 0.001.

We next studied the regulation and function of BNP in skeletal muscle *in vivo*. As shown in Fig. 3D, muscle-specific overexpression of PGC-1α in mouse muscle strongly promoted BNP gene expression. Then, we assessed the potential of BNP to modulate the activation of tissue-resident macrophages in muscle by injecting recombinant BNP into TA muscles of WT animals (or Pcolce and PBS as negative and vehicle control, respectively). Recapitulating the *in vitro* data, BNP injection increased the expression of TNFα, IL-6, MIP-1α, MCP-1 and of all tested M2 cytokines (Fig. 6A and B). In contrast, IL-12 expression was unaffected by BNP treatment, which reinforces the hypothesis of IL-12 to be inhibited by PGC-1α in muscle cells. As observed *in vitro*, Pcolce did not have any effect on M1 or M2 cytokine expression levels. Notably, the chemokines identified in the CM were also strongly elevated in the BNP-injected TA (Fig. 6C), implying a central role for BNP in the hierarchical control of chemokine expression in muscle.

Finally, we investigated whether exercise, a physiological context of PGC-1α induction, also increases the expression of BNP in skeletal muscle *in vivo*. For that purpose, an exhaustion exercise test was performed and BNP levels analyzed in quadriceps muscle. In exercised animals, the induction of PGC-1α gene expression was paralleled by an increase in BNP transcript levels immediately post-exercise (Fig. 6D). While BNP expression was reduced to baseline levels 4 h post-exercise, PGC-1α levels further increased and possibly activated other gene programs. Intriguingly, the chemokines CCL7, CXCL1 and CXCL10, which were identified in the CM of C2C12 myotubes overexpressing PGC-1α, also exhibited a time-dependent elevation post-exercise (Fig. 6E). Since the activity of M1 and M2 macrophages is tightly coordinated for proper muscle repair and regeneration post-exercise, we also determined how the expression levels of M1 and M2 cytokines were regulated immediately and 4 h after exercise cessation (Fig. 6F). Immediately after exercise, TNFα, IL-6, TGFβ and IL-10 were also elevated. The expression of the cytokines that were induced immediately after exercise dropped back to baseline levels after 4 h, while the expression of MIP-1α, MCP-1 and CCL22 only started to rise at this later time point. This time course experiment demonstrates that several M1 and M2 cytokines are differentially induced in response to exercise, and only some follow the immediate early transcription pattern of PGC-1α, most notable BNP.

## Discussion

Exercise, muscle regeneration and metabolism are intrinsically linked to inflammatory events. We now demonstrate that muscle PGC-1α, a key regulator of skeletal muscle plasticity in endurance training, not only modulates inflammation in muscle fibers, but also in tissue-resident immune cells by controlling paracrine myokine signaling. Interestingly, a PGC-1α-dependent reduction in muscle cell inflammation was not only shown in previous experiments in cultured muscle cells^10^, but is further highlighted by the specific and strong inhibition of IL-12 in muscle fibers observed in the present study. In addition to the cell autonomous effects, muscle PGC-1α triggers a paracrine signaling to activate tissue-resident macrophages. Importantly, even though a strong anti-inflammatory pattern of cytokine expression representing an M2-type polarization is observed, the less robust increase in M1, pro-inflammatory cytokine suggests that muscle PGC-1α could generally prime macrophages for rapid action, in which an M1 to M2 transition has to be tightly orchestrated to ensure proper repair and regeneration. An increased regenerative capacity in muscles overexpressing PGC-1α has recently been postulated^15^. We have shown that in addition to the increase of PGC-1α expression immediately and 4 h post-exercise, the expression of several chemokines and cytokines was also elevated 0 h and 4 h after exercise. The time-dependent changes in expression levels of these cytokines suggest that tissue macrophages are activated immediately after an acute bout of exercise. However, the exact time course of macrophage infiltration in response to endurance exercise is not well established. Interestingly, sustained overexpression of PGC-1α in muscle stimulates macrophage infiltration, as demonstrated by the higher number of tissue-resident macrophages in muscles of MCKα mice with a predominant M2 phenotype^16^,^17^. Muscle PGC-1α could thereby be indicative of the higher resistance of trained muscle fibers against damage and the more efficient repair and regeneration process^18^,^19^ in a physiological context with consistently elevated PGC-1α gene expression. Indeed, the higher proliferative rate of satellite cells in MCKα mice further supports this hypothesis^15^. However, the exact chain of events will have to be elucidated in a temporal analysis in animal models *in vivo* after acute exercise and chronic training that both reflect the pulsatile induction of PGC-1α after each exercise bout and the persistent increase in basal PGC-1α expression linked to the fiber-type switch after prolonged endurance training^20^.

PGC-1α regulates a number of myokines in skeletal muscle^7^, at least some of which were not able to activate cultured macrophages. Based on an unbiased secretome analysis of conditioned media obtained from cultured skeletal muscle cells overexpressing PGC-1α, candidate proteins were tested and BNP identified as a strong mediator of macrophage activation, both in terms of M1 and M2 prototypical cytokine expression. BNP is mainly known to be synthesized and secreted by cardiac ventricles in response to stretch in order to initiate signaling to reduce blood pressure and blood volume^21^,^22^,^23^. The administration of BNP can be used to treat patients with heart failure to reduce additional stress and possibly also fibrosis in the heart^24^. Importantly however, BNP also affects non-cardiac tissue, such as the liver, fat and skeletal muscle tissue^25^,^26^,^27^,^28^. Similar to the observations in the heart, transgenic overexpressing of BNP is able to reduce liver fibrosis^28^, indicating a more general role for BNP in inflammation linked to tissue repair and regeneration. An immunomodulatory role of BNP in macrophages is further implied by experiments demonstrating that stimulation of THP-1 macrophages with BNP results in increased ROS, NO_2_ and leukotriene B4 production^29^. Notably, besides the pro-inflammatory effects, the anti-inflammatory prostaglandin 2 and IL-10 levels were also increased by BNP in this context^29^, which is reminiscent of our findings in skeletal muscle. Our data now suggest that the PGC-1α-induced elevation of BNP plays a role in the cross-talk between skeletal muscle and macrophages and could thereby also influence inflammation, repair and regeneration of this tissue after exercise.

In a different study, the secreted phosphoprotein 1 (SPP1) was identified as a mediator of muscle PGC-1α-triggered macrophage activation leading to endothelial remodeling and neovascularization^17^. Curiously, SPP1 was not found in our mass spectrometric secretome analysis. It is unclear whether the divergent results are based on differences in the experimental models or if engagement of BNP and SPP1 might be context-dependent and geared towards macrophage activation in exercise-linked repair and regeneration vs. vascularization, respectively. However, BNP and SPP1 seem to have a similar effect on macrophages, as treatment of macrophages with BNP or SPP1 both increased the expression of MCP-1. It is obviously conceivable that both myokines are induced by muscle PGC-1α in a redundant manner to ensure robust activation of immune cells.

**Figure 7:**
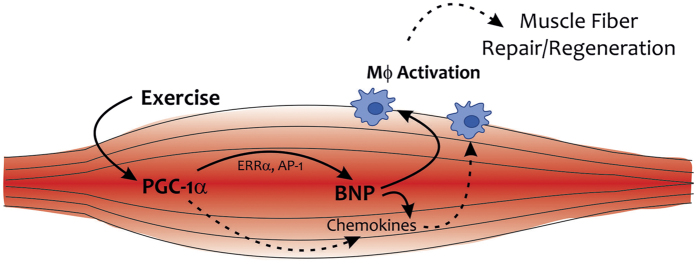
Schematic representation of the exercise- and PGC-1α-induced cross-talk between muscle and macrophages. PGC-1α increases BNP expression by co-activating ERRα and/or AP1. BNP subsequently induces a chemokine cocktail in muscle fibers, and activates macrophages in a local, paracrine manner, which then may contribute to the enhanced repair and regeneration potential of trained muscles.

Furthermore, BNP was shown to induce a browning phenotype and enhanced lipolysis in cultured adipocytes^26^,^30^, increase energy expenditure^26^, as well as to reduce the increase in fat mass in response to a high fat diet (HFD) in mice^27^. In addition, chronic BNP infusion improved glucose tolerance and insulin signaling in HFD-fed and obese mice, respectively due to a reduction in lipotoxicity within the muscle^31^. Therefore, it is not surprising that BNP levels are inversely correlated with BMI^32^, indicating that the possible protective effects of BNP against obesity and diabetes are blunted in obese individuals with lower circulating BNP^33^. This large variety of effects of BNP suggests that BNP plays a role in mediating some of the health beneficial effects observed in response to exercise. In humans, plasma BNP levels are increased immediately after exercise^34^,^35^. Due to the much higher expression of BNP in cardiac compared to skeletal muscle, the bulk of the circulating BNP could originate from the heart. However, 8 hours post-exercise, mRNA expression of the natriuretic peptide receptor A (NPR-A) is elevated in skeletal muscle^25^. Moreover, a myokine named musclin was recently described to be induced by exercise and to activate the same pathway as natriuretic peptides^36^. In particular, due to the binding of musclin to the clearance receptor NPR-C, plasma levels of NPs remain higher after exercise and can thereby elicit their positive effects on whole body metabolism in a more potent manner^36^. Intriguingly, musclin function has also been linked to exercise-induced PGC-1α gene expression in skeletal muscle. Together, these results underline the significance of a PGC-1α-BNP axis in paracrine signaling in exercised skeletal muscle that we postulate. Based on our findings and those published previously, it is conceivable that BNP is one important component of a larger cocktail of PGC-1α-controlled myokines with paracrine immunomodulatory function in skeletal muscle (Fig. 7). Whether a link between PGC-1α and BNP in muscle has systemic consequences remains to be elucidated. However, such a regulation might also occur in cardiac tissue with high PGC-1α expression and could thereby be important for the metabolic effects of BNP on liver, adipose tissue and potentially other metabolic organs.

In conclusion, we now describe a novel regulation and function of BNP in exercised skeletal muscle to activate tissue-resident macrophages when induced by PGC-1α and ERRα. Collectively, our results highlight a direct mechanism by which skeletal muscle fibers engage tissue macrophages after contraction. This novel fundamental pathway illustrates how muscle fibers orchestrate macrophage activation and thereby also provides an explanation for the enhanced capacity of trained skeletal muscle to respond to contractile damage. Importantly, this active engagement of immune cells by muscle fibers not only is of high importance for exercise adaptation, but could also be leveraged in the many different muscle diseases that are associated with aberrant inflammation. This axis could be of therapeutic importance in the treatment of muscle wasting diseases to accelerate and improve fiber repair and muscle regeneration.

## Methods

### Mouse experiments

C57BL/6 mice expressing PGC-1α^9^ (MCKα) under the control of the muscle creatine kinase (MCK) promoter were bred with respective WT mice to obtain WT and transgenic littermates. Male mice were maintained on a standard rodent chow with 12 h light/dark cycle and euthanized at 12 weeks of age when quadriceps muscles were collected for further analysis. Animals within the injection experiment were 15–18 weeks of age. 400ng of BNP (Cloud-Clone Corp) or Pcolce (R&D Systems) in 30 μl PBS were injected *i.m.* into the tibialis anterior (TA) and PBS as a control on the contralateral side. After 4 h, animals were euthanized and TAs collected for further analysis. Animals performing exhaustion exercise were at the age of 20 weeks. The incremental exercise at a 5° inclination started at a velocity of 10 m/min and was increased every 15 min by 2 m/min until exhaustion. Tissue collection was performed immediately after or 4 h after exercise. All methods were carried out in accordance with relevant guidelines and regulations, and all animal experiments were approved by the Kantonales Veterinäramt Basel-Stadt.

### Cell culture

The mouse myoblast cell line C2C12 was maintained below confluence in Dulbecco’s Modified Eagle Medium (DMEM) supplemented with 10% fetal calf serum and 1x Penicillin/Streptomycin (Invitrogen). For differentiation into myotubes, growth medium was exchanged for DMEM supplemented with 2% horse serum (Invitrogen) for 3–4 days. PGC-1α or GFP were overexpressed from recombinant adenoviral constructs 48 h prior to harvesting the cells. 24 hours after infection the medium was exchanged for differentiation medium. The knock-down experiment is described elsewhere^12^.

Conditioned medium was obtained from mouse C2C12 myotubes. After 3–4 days of differentiation, myotubes were infected with PGC-1α or GFP using a recombinant adenoviral construct for 24 h. Infection medium was exchanged after 24 h, which was exchanged again after another 24 h for differentiation medium that was used for the conditioned medium. The conditioned medium was removed after 20 h, filtered and stored at −20 °C.

The mouse RAW 264.7 macrophage cell line was maintained in DMEM supplemented with 10% fetal calf serum and 1x Penicillin/Streptomycin. At 80–90% confluence, the RAW macrophages were treated with conditioned medium of C2C12 myotubes for 4 h and then dissolved in Trizol for RNA extraction. RAW macrophages were treated for 4 h with 100 ng/ml irisin (AdipoGen), 10 ng/ml IL-15 (R&D Systems), 100 ng/ml BDNF (R&D Systems), 100 ng/ml CXCL1 (R&D Systems), 100 ng/ml CXCL2 (R&D Systems), 100 ng/ml CXCL5 (R&D Systems), 100 ng/ml LCN2 (Cell Signaling Technology), 200 ng/ml Pcolce (R&D Systems) or 1 nM, 2 nM or 4 nM BNP (Cloud-Clone Corp). After the 4 h treatment, the cells were dissolved in Trizol for RNA extraction.

### Semi-quantitative real-time PCR

Muscle tissue was homogenized using matrix particles (Qbiogene) in a FastPrep FP120 cell disrupter (Thermo Scientific) and RNA isolated with Trizol (Invitrogen) while residual DNA contamination was removed by DNase I (Invitrogen) digestion. 1 μg of RNA was reverse transcribed with SuperScript II (Invitrogen) and the resulting cDNA used as template for RT-PCR. To detect relative expression levels cDNA was amplified with the SYBR Green Master mix (Applied Biosystems) and analyzed on a StepOnePlus RT-PCR System (Applied Biosystems). The respective primer pairs are listed in the Supplemental Table S1. All values were normalized to the expression of TATA-Box binding protein (TBP) and expressed as fold induction over GFP-expressing control cells or WT animals.

### Proteomics analysis

For mass spectrometry analyses, serum-free medium was conditioned as described above. Serum-free CM had the same effect on RAW macrophages concerning M1 and M2 cytokine expression as CM with serum (data not shown).

Supernatants of 3 independent experiments containing the secreted proteins were collected and concentrated using ultrafiltration 5 kDa cut-off cartridges (Sartorius, VIVASPIN 500) according to the manufacture instructions. Protein samples were washed twice with 400 μl digestion buffer (1 M urea, 0.1 M ammoniumbicarbonate) and concentrated to a final volume of 100 μl. Proteins were reduced with 5 mM TCEP for 60 min at 37 °C, alkylated with 10 mM iodoacetamide for 30 min in the dark at room temperature and digested with trypsin (Promega) at 37 °C overnight (protein to trypsin ratio: 50:1). After digestion, the samples were supplemented with TFA to a final concentration of 0.5%. Then, peptides were desalted on C18 reversed phase spin columns according to the manufacturer’s instructions (Macrospin, Harvard Apparatus), dried under vacuum and stored at −80 °C until further processing.

1 µg of peptides of each sample were subjected to LC–MS analysis using a dual pressure LTQ-Orbitrap Velos mass spectrometer connected to an electrospray ion source (both Thermo Fisher Scientific) as described recently with a few modifications^37^. In brief, peptide separation was carried out using an EASY nLC-1000 system (Thermo Fisher Scientific) equipped with a RP-HPLC column (75 μm × 30 cm) packed in-house with C18 resin (ReproSil-Pur C18–AQ, 1.9 μm resin; Dr. Maisch GmbH, Ammerbuch-Entringen, Germany) using a linear gradient from 95% solvent A (0.15% formic acid, 2% acetonitrile) and 5% solvent B (98% acetonitrile, 0.15% formic acid) to 28% solvent B over 120 min at a flow rate of 0.2 μl/min. The data acquisition mode was set to obtain one high resolution MS scan in the FT part of the mass spectrometer at a resolution of 60,000 full width at half-maximum (at m/z 400) followed by MS/MS scans in the linear ion trap of the 20 most intense ions using rapid scan speed. The charged state screening modus was enabled to exclude unassigned and singly charged ions and the dynamic exclusion duration was set to 30 s. The ion accumulation time was set to 300 ms (MS) and 25 ms (MS/MS).

For label-free quantification, the generated raw files were imported into the Progenesis LC-MS software (Nonlinear Dynamics, Version 4.0) and analyzed using the default parameter settings. MS/MS-data were exported directly from Progenesis LC-MS in mgf format and searched against a decoy database the forward and reverse sequences of the predicted proteome from *mus musculus* (UniProt, download date: 22/05/2013, total of 33,984 entries) using MASCOT (version 2.4.). The search criteria were set as follows: full tryptic specificity was required (cleavage after lysine or arginine residues); 3 missed cleavages were allowed; carbamidomethylation (C) was set as fixed modification; oxidation (M) as variable modification. The mass tolerance was set to 10 ppm for precursor ions and 0.6 Da for fragment ions. Results from the database search were imported into Progenesis LC-MS and the final peptide measurement list containing the peak areas of all identified peptides, respectively, was exported. This list was further processed and statistically analyzed using our in-house developed SafeQuant R script to obtain protein relative abundances^37^. This analysis included global data normalization by equalizing the total MS1 peak areas across all LC-MS runs, summation of MS1 peak areas per protein and LC-MS/MS run, followed by calculation of protein abundance ratios. The summarized protein expression values were used for statistical testing of between condition differentially abundant proteins. Here, empirical Bayes moderated t-Tests were applied, as implemented in the R/Bioconductor limma package. The resulting per protein and condition comparison p-values were adjusted for multiple testing using the Benjamini-Hochberg method. The peptide and protein false discovery rate (FDR) was set to 1% using the number of reverse hits in the dataset. All quantified proteins together with their identification and quantification details are listed in Supplemental Table S2.

### Data analyses and binding site prediction

Using ChIP-Seq data on PGC-1α occupancy in skeletal muscle cells from a previous study^12^, a 1.2 kilo base (kb) long region located ~7 kb upstream of the BNP gene (on chromosome 4 from 147’352’500 to 147’353’700 base pairs (bp), mm9 genome assembly) was identified as highly enriched in PGC-1α IP reads. In order to account for the conservation of transcription factor binding sites (TFBSs) across related species, this 1.2 kb long region was aligned, using the software T-Coffee^38^, to its orthologous regions from 6 mammalian species: human (hg18), opossum (monDom4), dog (canFam2), rhesus macaque (rheMac2), horse (equCab1) and cow (bosTau3). The software MotEvo^39^ was used on these alignments to predict TFBSs for a collection of 190 mammalian regulatory motifs that was downloaded from the SwissRegulon database^40^.

### Statistical analysis

Data were analyzed with either one-way ANOVA followed by Bonferroni post-hoc analyses or Student’s t test. *P* < 0.05 was considered statistically significant. Data are presented as mean ± SEM.

## Additional Information

**How to cite this article**: Furrer, R. *et al*. Paracrine cross-talk between skeletal muscle and macrophages in exercise by PGC-1α-controlled BNP. *Sci. Rep.* **7**, 40789; doi: 10.1038/srep40789 (2017).

**Publisher's note:** Springer Nature remains neutral with regard to jurisdictional claims in published maps and institutional affiliations.

## Supplementary Material

Supplementary Figures and Tables

Supplementary Dataset 1

## Figures and Tables

**Table 1:** Candidate proteins that were increased in the conditioned medium of cultured C2C12 myotubes overexpressing PGC-1α or GFP (See Table S1 for details).

Gene symbol	Gene name	Number of peptides used for quantification	Fold-change protein PGC-1α/GFP	q-value protein PGC-1α/GFP
CCL2/MCP-1	C-C motif chemokine 2	2	42.38***	0.015
CCL7	C-C motif chemokine 7	6	20.18***	0.028
CXCL1	C-X-C motif chemokine 1	4	69.99***	0.015
CXCL5	C-X-C motif chemokine 5	6	26.34***	0.015
CXCL10	C-X-C motif chemokine 10	2	62.43**	0.102
LCN2	Neutrophil gelatinase-associated lipocalin	1	115.45***	0.025
Pcolce	Procollagen C-endopeptidase enhancer 1	22	5.22**	0.110
Nppb/BNP	Natriuretic peptides B	2	10.62**	0.146

**p < 0.01; ***p < 0.001.
