# Supplementary material for: Paracrine cross-talk between skeletal muscle and macrophages in exercise by PGC-1α-controlled BNP
Source: Sci Rep. 2017 Jan 16;7:40789. doi: 10.1038/srep40789 (PMC5238507; doi:10.1038/srep40789)
Supplement: Supplementary Figures and Tables [file srep40789-s1.pdf]

Paracrine cross-talk between skeletal muscle and macrophages in exercise by PGC-1α-controlled BNP

Regula Furrer[1,%], Petra S. Eisele[1,%,&], Alexander Schmidt[2], Markus Beer[1,#], and Christoph Handschin[1,*]

[1]Biozentrum, Division of Pharmacology/Neurobiology, University of Basel,
CH-4056 Basel, Switzerland
[2]Biozentrum, Proteomics Core Facility, University of Basel,
CH-4056 Basel, Switzerland

[%]shared first authorship
[&]Present address: Labor Team W, Goldach, Switzerland
[#]This manuscript is dedicated to the memory of our coworker and friend Markus Beer (deceased on Aug 24, 2015).

[*]Materials & Correspondence: Christoph Handschin, Biozentrum, University of Basel, Klingelbergstrasse 50/70, CH-4056 Basel, Switzerland, Phone: +41 61 267 2378, Fax +41 61 267 2208, Email: christoph.handschin@unibas.ch

Supplemental Information

Supplemental Figures

Supplemental Figure S1 related to Figure 2. *Treatment of RAW macrophages with myokines induced by PGC-1α only mildly increased the expression of pro- and anti-inflammatory cytokines.* **A.** mRNA expression levels of myokines were measured in C2C12 myotubes adenovirally overexpressing PGC-1α or GFP **B, C.** RAW macrophages were treated for 4h with myokines known to be induced by PGC-1α. Relative expression of pro- (**B**) and anti-inflammatory (**C**) cytokines was determined by RT-PCR. Values represent the mean of at least 3 independent experiments +SEM. * P<0.05; ** P<0.01; *** P<0.001; myokine versus control.

Supplemental Figure S2 related to Figure 4. *Treatment of RAW macrophages with different BNP concentrations increased the expression of pro- and anti-inflammatory cytokines in a dose-dependent manner.* After treating RAW macrophages with PBS, 1, 2, and 4 nM BNP for 4 h, mRNA expression levels of pro- and anti-inflammatory cytokines were determined by RT-PCR. Values represent the mean of at least 3 independent experiments +SEM. * P<0.05; ** P<0.01; *** P<0.001; BNP treated versus control.

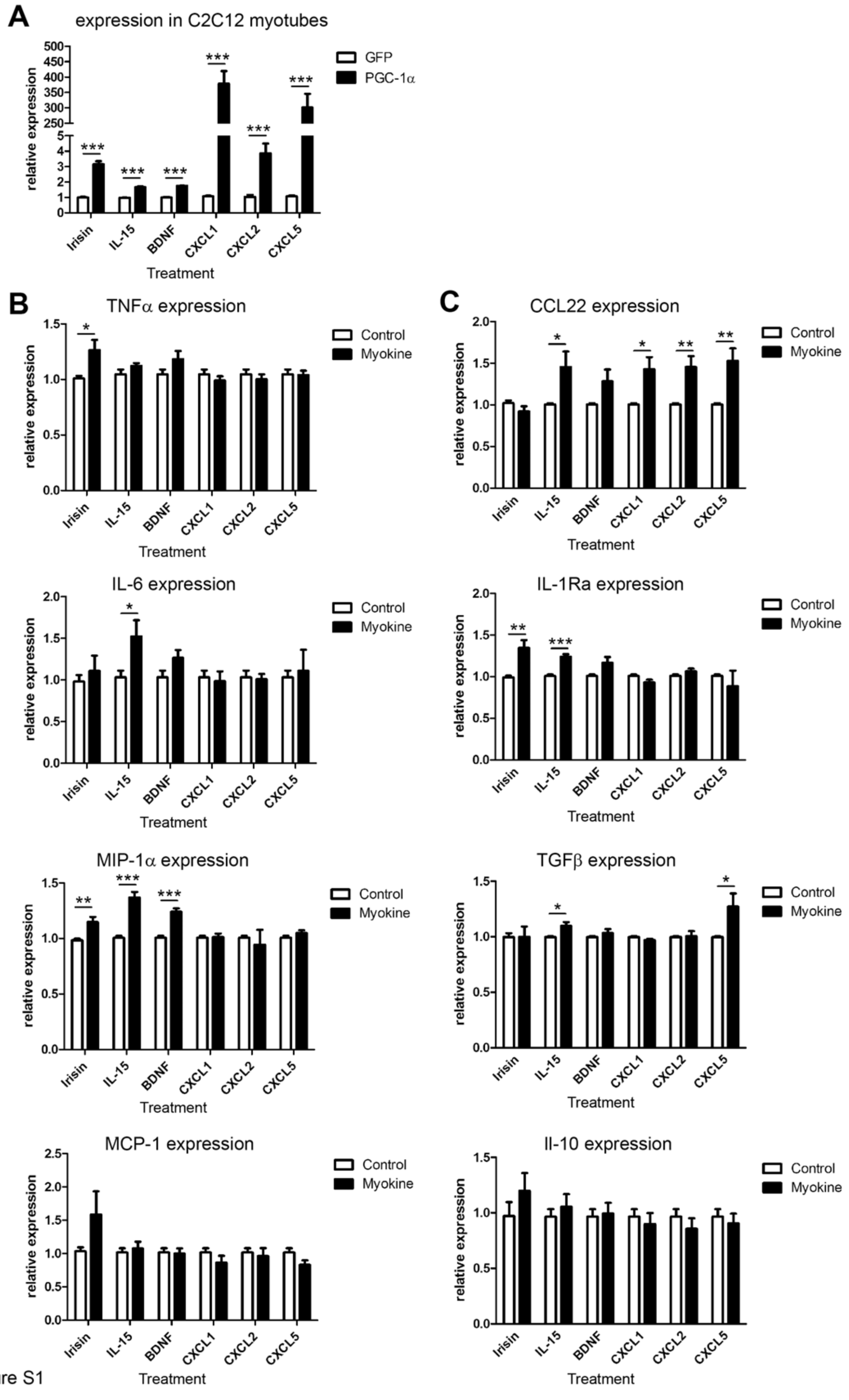
A
expression in C2C12 myotubes
GFP
PGC-1α
relative expression
Irisin
IL-15
BDNF
CXCL1
CXCL2
CXCL5
Treatment
B
TNFα expression
IL-6 expression
MIP-1α expression
MCP-1 expression
C
CCL22 expression
IL-1Ra expression
TGFβ expression
Il-10 expression
Control
Myokine

Figure S1

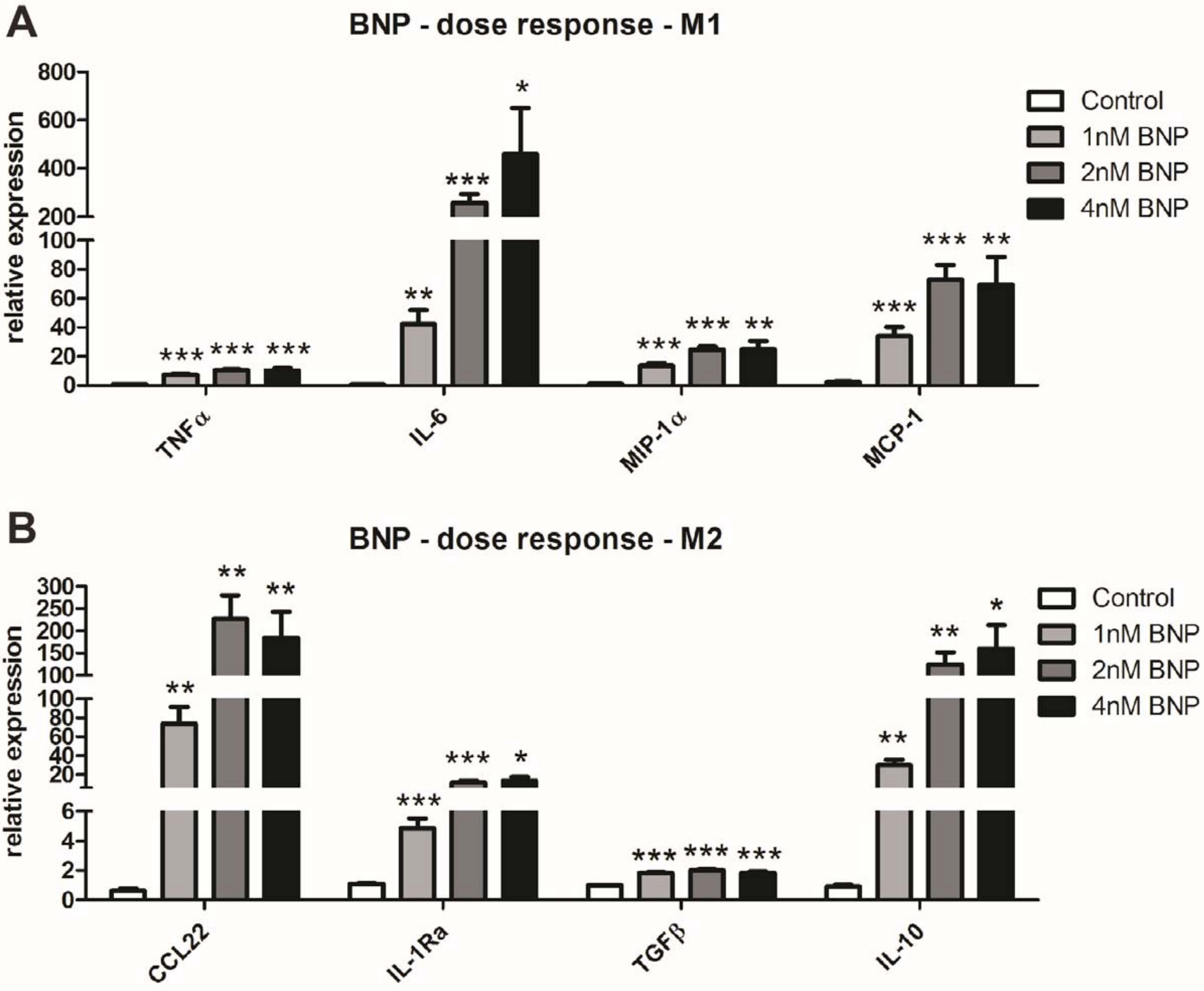
A
BNP - dose response - M1
relative expression
800
600
400
200
100
80
60
40
20
0
*
***
**
***
***
***
***
***
***
**
***
**
Control
1nM BNP
2nM BNP
4nM BNP
TNFα
IL-6
MIP-1α
MCP-1
B
BNP - dose response - M2
relative expression
300
250
200
150
100
100
80
60
40
20
6
4
2
0
**
**
**
**
***
***
*
***
***
***
**
**
*
Control
1nM BNP
2nM BNP
4nM BNP
CCL22
IL-1Ra
TGFβ
IL-10

Figure S2

Supplemental Tables

Supplemental Table S1: Primer sequences for RT-qPCR.

Gene	Forward primer	Reverse primer
**BDNF**	CGGCGCCCATGAAAGAAGTA	AGACCTCTCGAACCTGCCCT
**BNP**	GGCCTCACAAAAGAACACCC	TGCCCAAAGCAGCTTGAGAT
**CCL7**	GCTGCTTTCAGCATCCAAGTG	CCAGGGACACCGACTACTG
**CCL22**	TGGAGTAGCTTCTTCACCCA	TCTGGACCTCAAAATCCTGC
**CXCL1**	TGAGCTGCGCTGTCAGTGCCT	AGAAGCCAGCGTTCACCAGA
**CXCL2**	GAGCTTGAGTGTGACGCCCCCAGG	GTTAGCCTTGCCTTTGTTCAGTATC
**CXCL5**	GCATTTCTGTTGCTGTTCACGCTG	CCTCCTTCTGGTTTTTCAGTTTAGC
**CXCL10**	TCTGAGTGGGACTCAAGGGA	AGGCTCGCAGGGATGATTTC
**IL-1Ra**	AAATCTGCTGGGGACCCTAC	TGAGCTGGTTGTTTCTCAGG
**IL-6**	CCACGGCCTTCCCTACTTC	TTGGGAGTGGTATCCTCTGTGA
**IL-10**	CTGGACAACATACTGCTAACCG	GGGCATCACTTCTACCAGGTAA
**IL-12**	GCTTCTCCCACAGGAGGTTT	CTAGACAAGGGCATGCTGGT
**IL-15**	GAGGCCAAGAAGAGTTCTGGAT	TGCCCAGGTAAGAGCTTCAA
**Irisin**	ATGAAGGAGATGGGGAGGAA	GCGGCAGAAGAGAGCTATAACA
**LCN2**	TCTGATCCAGTAGCGACAGC	CAGAAGGCAGCTTTACGATG
**MCP-1**	CCCAATGAGTAGGCTGGAGA	TCTGGACCCATTCCTTCTTG
**MIP-1α**	TCCCAGCCAGGTGTCATTTT	TTGGAGTCAGCGCAGATCTG
**Pcolce**	GCTCCATATCGAAGACTCGG	GTTACGTGGCAAGTGAGGGT
**PGC-1α**	TGATGTGAATGACTTGGATACAGACA	GCTCATTGTTGTACTGGTTGGATATG
**TBP**	GGCCTCTCAGAAGCATCACTA	GCCAAGCCCTGAGCATAA
**TGFβ**	CAACCCAGGTCCTTCCTAAA	GGAGAGCCCTGGATACCAAC
**TNFα**	CACAAGATGCTGGGACAGTGA	TCCTTGATGGTGGTGCATGA

Supplemental Table S2: Complete proteomic analysis

See attached Excel file "Proteomics_Supplemental_Table_S1.xlsx"
